# Factors associated with asthma among under-fives in Mulago hospital, Kampala Uganda: a cross sectional study

**DOI:** 10.1186/1471-2431-13-141

**Published:** 2013-09-11

**Authors:** Rebecca Nantanda, Marianne S Ostergaard, Grace Ndeezi, James K Tumwine

**Affiliations:** 1 Child Health and Development Centre, Makerere University College of Health Sciences, Kampala, Uganda; 2 Department of Public Health, Section of General Practice, University of Copenhagen, Copenhagen, Denmark; 3 Department of Paediatrics and Child Health, Makerere University College of Health Sciences, Kampala, Uganda

**Keywords:** Acute respiratory symptoms, Asthma, Associated factors, Under-five, Prevalence, Resource limited settings

## Abstract

**Background:**

Asthma is the most common chronic childhood illness, with rapidly increasing prevalence in low-income countries. Among young children, asthma is often under-diagnosed.

We investigated the factors associated with asthma among under-fives presenting with acute respiratory symptoms at Mulago hospital, Uganda.

**Methods:**

A hospital-based cross sectional study of 614 children with cough and/or difficult breathing, and fast breathing, was conducted between August 2011 and June 2012. A questionnaire focusing on clinical history of the child was administered to the caretakers. A physical examination and, laboratory and radiological investigations were done. Asthma was defined according to GINA (Global Initiative for Asthma) guidelines which were modified by excluding the symptom of “chest tightness”, spirometry/peak expiratory flow measurements and by adding chest x-ray findings to distinguish asthma from pneumonia. A panel of three paediatricians reviewed the participants’ case reports and, guided by the study definitions, made a diagnosis of asthma or other. Multivariable logistic regression analysis was done to determine factors independently associated with asthma.

**Results:**

Of the 614 children, 128 (20.8%) had asthma, 125 (20.4%) bronchiolitis, 167 (27.2%) bacterial pneumonia only, 163 (26.5%) viral pneumonia while 31 (5.1%) had other diagnoses including pulmonary tuberculosis. The majority (71.1%) of children with asthma were aged ≥ 12 months. Factors associated with asthma included maternal asthma (AOR 2.4, 95% CI 1.2, 4.6), a history of allergy in the patient (AOR 2.6, 95% CI 1.2, 5.4,), use of gas for cooking (AOR 3.8, 95% CI 1.2, 13.3), prematurity (AOR 9.3, 95% CI 1.2, 83.3) and high level of education of caretaker (AOR 9.1, 95% CI 1.1, 72.8).

**Conclusion:**

Maternal asthma, a history of allergy in the patient, use of gas for cooking, prematurity and high level of education of caretaker were significantly associated with asthma. There is need for studies to explore the role of the above factors in development and exacerbation of childhood asthma to provide information that can be used to design strategies for asthma prevention and control.

## Background

Asthma is the most common chronic childhood condition world-wide. Its prevalence is highest in industrialized countries and rapidly increasing in low-income countries [1,2]. In Africa, the majority of studies on childhood asthma were carried out among children aged 7-14 years [1-4]. Although evidence suggests that asthma starts in infancy, few researchers have studied risk factors for asthma in young children [5]. Hitherto, factors associated with asthma among children less than five years have not been established in Uganda.

In Africa, studies have revealed factors associated with asthma that can guide clinicians in asthma case detection and management [2,5]. In these studies, childhood asthma has been associated with genetic factors such as a family history of asthma and allergy as well as male gender. Environmental factors such as in-door and out-door allergens, air pollution, second hand smoking and respiratory tract infections have also been linked to childhood asthma [5-7]. Most of these studies were in developed countries, yet, asthma phenotypes and, the role and extent of the factors associated with asthma differ in developed and low-income countries [8,9]. Research has also indicated that, the role of some factors associated with asthma such as exclusive breastfeeding and early exposure to infection is controversial [10-12].

In young children, acute asthma and bronchiolitis have similar clinical presentation [13], and it is sometimes difficult to distinguish asthma from bronchiolitis in this age group. Studies in industrialized nations have indicated similarities between factors associated with asthma and bronchiolitis [14,15]. Information regarding factors associated with bronchiolitis in Uganda is lacking.

The main objective of the current study was to describe the factors associated with asthma among children less than five years who presented with acute respiratory symptoms in a tertiary hospital in Uganda. The secondary objective was to draw comparisons between factors associated with asthma and bronchiolitis. We hypothesized that factors associated with bronchiolitis and asthma among under-fives are similar. Understanding factors associated with asthma among children less than five years in low-income settings has the potential to improve on asthma case detection, adaptation of preventive measures and better asthma management, and this may lead to better quality of life of the affected children.

## Methods

### Design and setting

We conducted a cross-sectional study among children aged 2 to 59 months presenting at the emergency paediatric unit of Mulago hospital Kampala between August 2011 and July 2012. Mulago hospital is a national referral hospital. It also acts as a district hospital serving an urban and peri-urban catchment population of about two million people. The paediatric emergency unit attends to children aged 1 day to 12 years. The average daily attendance is 80 children, 75% of whom are aged 2 to 59 months. An estimated 25% of the children present with cough and/or difficulty in breathing. The hospital was selected as the study site because of its ability to handle laboratory and radiological investigations for diagnosis of asthma and pneumonia, facilities that are not readily available in rural Ugandan hospitals.

The study was approved by the Higher Degrees, Ethics and Research Committee (HDREC) at Makerere University College of Health Sciences and the Uganda National Council of Science and Technology. Informed written consent was obtained from the caretakers of the participants.

### Development of the questionnaire

The process of questionnaire development was led by one of us (MSO) in Denmark. Through a literature search, research team debates and expert opinion, study definitions and concepts for asthma were discussed. A qualitative study, focusing on caretakers of children with asthma, was undertaken to identify relevant items to be included in the questionnaire. The interviews were aimed at understanding the presentation and progression of the disease from the caretaker’s perspective. A preliminary questionnaire was hence developed consisting of items focusing on acute symptoms, past medical history of the child and medicine use during previous illnesses and, family history of asthma. The items were then tested for language, understanding and relevance through focus group discussions with caretakers. A new questionnaire was developed and this was further tested twice in the same way and a final version was generated, which was then tested for internal and external validity. It was then translated into English.

The questionnaire was then adapted for use in Uganda by one of us (RN). It was translated to *Luganda*, the language commonly used in central Uganda, where Mulago hospital is located. It was then back-translated into English. Both the *Luganda* and English versions were pre-tested on a sample of 35 mothers to check for understanding of the questionnaire items and time taken to administer the questionnaire. Any necessary changes were made and a final questionnaire was developed.

### Participants and recruitment

We enrolled children aged 2 to 59 months who presented at the paediatric emergency unit of Mulago hospital with cough and/or difficulty in breathing plus fast breathing, and whose caretakers gave informed written consent. The definition of fast breathing was based on WHO criteria [16] Children with heart conditions, or cardiac failure secondary to severe anaemia, based on the caretaker’s history, physical examination findings and medical records, were excluded. All potential participants were triaged and those with ‘severe classification’ according to the WHO guidelines [17] were given urgent care before proceeding with the consent process. Children with wheezing were nebulised with salbutamol solution using an ultrasonic nebulizer according to the hospital protocol [18] and the response noted. Participants were enrolled from 8.00.am to 10.00.pm on weekdays.

After enrolment, a questionnaire (Additional file 1) was administered by the nurse. A physical examination was performed by the doctor. For all participants, we measured the peripheral oxygen saturation (SaO_2_) in room air. Children with SaO_2_ less than 92% were given oxygen by mask or nasal prongs. Six mill-litres of venous blood were drawn from the cubital vein or dorsum of the hand using a BD™ blood collection set in three aliquots; for blood culture, white cell count, and serum C-reactive protein (CRP) titres. A peripheral blood smear for malaria parasites was also done. A specimen of nasopharyngeal epithelium was collected for identification of Respiratory Syncytial Virus (RSV) according to the manufacturer’s instructions (BD Diagnostics, Becton, Dickinson and Company, Maryland USA). All specimens were delivered to the laboratory within six hours of collection. A posterior-anterior chest x-ray was taken for each of the study participants within 48 hours of enrolment.

### Laboratory and radiological investigations

Total and differential white blood cell counts were determined using Coulter counter method (Beckman Coulter Inc. Z™ series). CRP titres were analyzed using CRP (Human) ELISA kit-ABNOVA™, Taiwan, according to the manufacturer’s instructions. Blood culture was done using the Bactec method (Becton, Dickson and Company Maryland USA) and positive samples were further analyzed for the bacterial species using a Gram stain. Drug susceptibility tests were done using the Disc diffusion method [19]. For malaria diagnosis, a peripheral blood smear was prepared using Leishman’s stain. Identification of Respiratory Synctial Virus (RSV) from nasal epithelium was done using Direct Fluorescence Antibody (DFA) technique (Light Diagnostics™ USA). The x-rays were interpreted by two independent radiologists who were blinded to the clinical and laboratory findings of the participants. Radiographic end-points included consolidation, collapse, alveolar and interstitial infiltrates, pleural effusion, hyper-inflation and normal. X-rays with discordant results from the two radiologists were interpreted by a third reader and the result taken as final if there was concordance between the third reader and any of the primary readers.

### Definition of asthma and bronchiolitis

There are no diagnostic gold standards for asthma among under-fives. In this study, we modified the GINA (Global Initiative for Asthma) guidelines for diagnosis of asthma [20] as follows: In the history; we excluded “recurrent chest tightness” as a symptom because it is not easily expressed by children less than five years [21,22]. We also we excluded peak expiratory flow measurements because children less than five years are not able to perform this test effectively [23]. Furthermore, we included chest x-rays to help distinguish asthma from pneumonia. Pneumonia is common in Uganda and, in under-fives, has a presentation similar to that of acute asthma [13,24].

The case definition of bronchiolitis was based on South African guidelines [14] for diagnosis, management and prevention of acute viral bronchiolitis. The details of the study definitions are provided in Table 1.

**Table 1:** Case definitions for asthma, bronchiolitis and pneumonia

**Diagnostic category**	**Criteria**
***Asthma***	1) Cough, wheeze, difficulty in breathing ***(at least one)***
**Highly probable if 4 of 5 are present**	2) Recurrent cough, wheeze and/or difficulty in breathing, positive history of atopy in child (eczema, rhinitis, food, conjunctivitis), history of asthma in first degree relative ***(at least one)***
	3) Fast breathing, chest in drawing, prolonged expiration, rhonchi ***(at least 3)***
	4) Good response to bronchodilators
	5) Chest x-ray: normal or hyperinflation
***Bronchiolitis***	1) Age less than 2 years, cough, difficulty in breathing, index episode of wheeze ***(all must be present)***
Highly probable if 1 and any other criteria are present	2) Fast breathing, prolonged expiration, chest in-drawing, rhonchi (at least two)
	3) Total white cell count ≤15x10^9^cells/l, CRP < 40 mg/l, positive RSV ***(at least one)***
	4) Chest x-ray: normal or hyperinflation
***Bacterial pneumonia***	1) Fever, cough and or difficulty in breathing ***(at least two)***
**Highly probable if 4 of 5 are present**	2) Axillary temperature ≥38°C, fast breathing, chest indrawing ***(at least 2)***
	3) CRP 40 mg/l, total white cell count ≤ 15 ×10^9^ cells/l, Neutrophils ≥65% ***(at least one)***
	4) Positive blood culture
	5) Chest x-ray: alveolar infiltrates, consolidation, pleural effusion ***(at least one)***
***Viral pneumonia***	1) Fever, cough, difficulty in breathing ***(at least one)***
**Highly probable if 3 of 4 are present**	2) Axillary temperature ≥38°C, fast breathing, chest indrawing ***(at least 2)***
	3) CRP <40 mg/l, total white cell count <15 ×10^9^ cells/l, lymphocytes ≥45%, positive RSV ***(at least one)***
	4) Chest x-ray: normal or diffuse infiltrates

### Diagnosis of asthma

A panel of experts comprising paediatricians with experience in pulmonology and infectious diseases reviewed the participants’ case records. The experts had no access to the participants; hence the diagnoses were made post hoc. Each expert studied the case record of the participant, and guided by the study definitions, made a diagnosis, which was then discussed by all the panellists. A diagnosis of asthma or of some other condition such as pneumonia was made following agreement of all or two of the panellists. Where there was discordance between all the three panellists, the case records were subjected to a further discussion until a diagnosis was agreed upon. One of us (RN) took the minutes during the proceedings but did not participate in the discussions.

### Statistical considerations

To describe factors associated with asthma among young children with cough and/or difficult breathing, a minimum sample size of 308 was calculated. We assumed two-sided significance level of 95%, power of 80%, proportion of children with asthma and who had a family history of asthma to be 52%, and Odds’ ratio of 2.5, based on a study of asthma in preschoolers by Haby and colleagues [7]. However, this was part of a larger study involving 614 children and all were included in the analysis.

Data was double-entered into Epidata version 3.0 and exported to Stata version 12.0 (Stata Corp, College station Texas, USA) for analysis. To determine factors independently associated with asthma, multivariable analysis was done. A logistic regression model was built by including all factors with a p value less than 0.2 at bivariate analysis. Adjusted Odd’s ratios were computed to adjust for confounding. Multi-colinearlity and interaction of the predictor variables was checked until we obtained the best fitting model. Cohen’s kappa was used to measure the degree of agreement between the primary radiologists. A p value of ≤0.05 was considered statistically significant. We also performed logistic regression analysis for factors associated with bronchiolitis and compared them to those associated with asthma. Results are summarized as frequencies, proportions, figures and tables as appropriate.

In this study, children from the capital city, municipalities and town councils in Uganda were collectively referred to as coming from “urban setting” and the rest from “rural setting”. This was adapted from the Uganda Demographic and Health Survey [24].

## Results

### Demographic characteristics of the participants

Of the 986 children aged 2 to 59 months who presented with cough and/or difficulty in breathing during the study period, 614 were recruited. The remaining 372 children were not recruited because: 189 (50.8%) did not fulfill the inclusion criteria, 150 (40.3%) had caretakers who declined to participate and 33 (8.9%) died before any investigations could be done.

Of the 614 participants, 347 (56.5%) were male. The median age was 10 months (Inter-quartile range 6–18 months) and 54.2% (333 of 614) were less than 12 months of age. Seventy six percent (468 of 614) were from urban settings (Table 2). Chest x-rays were obtained in 590 (96.0%) of the 614 participants. The primary radiologists agreed on the chest x-ray findings in 79.4% of cases (Cohen’s kappa 0.72, p = 0.000).

**Table 2:** Baseline characteristics of the study participants (N = 614)

**Characteristic**		**Frequency (n)**	**Percentage (%)**
Age	< 12 months	333	54.2
	12–24 months	160	26.1
	>24 months	121	19.7
Sex	Male	347	56.5
Residence	Urban	468	76.2
Diagnoses	Asthma	78	12.7
	Bronchiolitis	125	20.4
	Combined asthma and bacterial pneumonia	50	8.1
	Bacterial pneumonia	167	27.2
	Viral pneumonia	163	26.5
	Others (e.g. PTB, PJP)	31	5.1
Genetic risk factors for asthma	Maternal asthma	60	9.8
	Allergy in patient	76	12.4
Environmental risk factors for asthma	Exposure to tobacco smoke	67	10.9
	Use of gas for cooking	12	2.0
	Lack of exclusive breastfeeding for at least 3 months	149	25.8
	High socioeconomic status of caretaker	39	6.4

PTB Pulmonary tuberculosis, PJP *Pneumocystis jirovecii* pneumonia.

Asthma was diagnosed in 128 (20.8%) of the total participants, 50 (39.1%) of whom had bacterial pneumonia as well. One hundred and twenty five children (20.4%) had bronchiolitis, 167 (27.2%) had bacterial pneumonia only, 163 (26.5%) had viral pneumonia while 31 (5.1%) had other diagnoses including pulmonary tuberculosis. Of the 128 children with asthma, 91 (71.1%) were aged 12 months and above and 71 (55.5%) were male. Only 24 (18.8%) of the children with asthma had been previously diagnosed.

### Factors associated with asthma

#### Genetic factors

Maternal asthma (AOR 2.4, 95% CI 1.2, 4.6, p = 0.009) and a history of allergy in the patient (AOR 2.6, 95% CI 1.2, 5.4, p = 0.015) were associated with asthma. There was no association between parental history of allergy and male gender, and asthma.

#### Environmental factors

We found a statistically significant association between asthma and use of gas for cooking (AOR 3.8, 95% CI 1.2–13.3, p = 0.035), prematurity (AOR 9.3, 95% CI 1.2–83.3, p = 0.044) and a high level of education of caretaker (AOR 9.1, 95% CI 1.1–72.9, p = 0.037). Although air pollution, in-door and out- door allergens and exposure to tobacco smoke have been previously associated with asthma, we did not find any significant association with them in this study. Similarly, there was no association between exclusive breastfeeding and asthma. Other factors studied included mode of delivery (vaginal versus Caesarean birth) and, birth weight. There was no association between the above factors and asthma (Table 3).

**Table 3:** Factors associated with asthma among children aged 2–59 months with acute respiratory symptoms in Mulago hospital, Uganda (N = 614)

**Variable**	**Asthma**		*COR (95% CI)	**†AOR (95% CI)**
	**Yes (%)**	**No (%)**		
***Patient factors***				
Maternal asthma:				
Yes	21.1	7.0	3.6 (2.0–6.6)	2.4 (1.2–4.6)
No	78.9	93.0		
History of allergy in patient:				
Yes	29.7	7.8	5.0 (2.9–8.5)	2.6 (1.2–5.4)
No	70.3	92.2		
Gender				
Male	55.5	56.8		
Female	44.5	43.2	1.1 (0.7–1.6)	
***Environmental factors***				
Exposure to tobacco smoke:				
Yes	14.1	10.1	1.5 (0.8–2.7)	1.5 (0.8–2.7)
No	85.9	89.9		
Use of gas for cooking:				
Yes	3.9	1.4	2.8 (0.7–10.4)	3.8 (1.2–13.3)
No	96.1	98.6		
Prematurity:				
Yes	1.6	5.3	3.6 (0.9–33.3)	9.3 (1.2–83.3)
No	98.4	94.7		
Exclusive breastfeeding for at least 3 months:				
Yes	76.5	66.3	1.7 (1.0–2.8)	1.5 (0.9–2.5)
No	23.5	33.7		
Education level of caretaker				
Primary	40.6	48.4	1.4 (0.9–2.1)	9.1 (1.1–72.8)
Post-primary	59.4	51.6		

*COR Crude Odds ratio, CI Confidence Interval,

†AOR Adjusted Odds ratio.

#### Factors associated with bronchiolitis

There was a statistically significant relationship between male sex and bronchiolitis (AOR 1.7, 95% CI = 1.1 –2.8, p = 0.011). There was marginal association between paternal history of asthma (AOR 6.6, 95% CI = 0.9–52.6, p = 0.070), a history of allergy in the study child (AOR 3.2, 95% CI = 0.9–11.1, p = 0.066) and bronchiolitis. There was no significant association between environmental factors such as air pollution, exposure to tobacco smoke and bronchiolitis (Table 4).

**Table 4:** Factors associated with bronchiolitis among children with acute respiratory symptoms in Mulago hospital, Uganda (N = 512)

**Variable**	**Bronchiolitis**		*COR (95% CI)	**†AOR (95% CI)**
	**Yes (%)**	**No (%)**		
***Patient factors***				
Maternal asthma				
Yes	4.8	9.1	2.0 (0.8–4.9)	1.9 (0.7–4.7)
No	95.2	90.9		
History of allergy in patient:				
Yes	4.8	11.1	2.5 (1.1–6.1)	2.1 (0.9–5.1)
No	95.2	88.9		
Gender:				
Male	68	52.2	1.9 (1.2–2.8)	1.9 (1.2–2.9)
Female	32	46.8		
***Environmental factors***				
Exposure to tobacco smoke:				
Yes	12	11.4	1.1 (0.6–2.0)	
No	88	88.6		
Use of gas for cooking				
Yes	0.8	2.6	3.3 (0.4–2.9)	
No	99.2	97.4		
Prematurity				
Yes	6.4	4.1	1.6 (0.7–3.8)	
No	93.6	95.9		
Exclusive breastfeeding for at least 3 months:				
Yes	75	73.6	4.2 (0.3–49.8)	
No	25	26.3		
Education level of caretaker:				
Primary	51.2	47.3	1.1 (0.8–1.4)	
Post–primary	48.8	52.7		

*COR Crude Odds ratio, CI Confidence Interval,

†AOR Adjusted Odds ratio.

#### Factors associated with asthma and bronchiolitis

We combined children with asthma and bronchiolitis and determined factors that were associated with this group of children. There was a statistically significant relationship between maternal history of asthma (AOR 2.0, 95% CI 1.1–3.8, p = 0.030), a history of allergy in the child (AOR 3.3, 95% CI 1.5–7.2, p = 0.004) and asthma or bronchiolitis. There was no significant association between environmental factors such as air pollution, exposure to tobacco smoke and, asthma and bronchiolitis (Table 5).

**Table 5:** Factors associated with asthma and bronchiolitis among children aged 2-59 months with acute respiratory symptoms in Mulago hospital, Uganda (N = 614)

**Variable**	**Asthma/Bronchiolitis**		*COR (95% CI)		**†AOR (95% CI)**	
	**Yes (%)**	**No (%)**				
***Patient factors***						
Maternal asthma						
Yes	13.8	6.9	2.2 (1.3–3.7)		2.0 (1.1–3.8)	
No	86.2	93.1				
History of allergy in patient:						
Yes	17.8	8.6	2.3 (1.4–3.7)		3.3 (1.5–7.2)	
No	82.2	91.4				
Gender:						
Male	61.7	52.9	1.4 (1.0–2.0)		1.1 (0.8–1.7)	
Female	38.3	47.1				
***Environmental factors***						
Exposure to tobacco smoke:						
Yes	13.0	9.4	1.4 (0.9–2.4)		1.3 (0.7–2.3)	
No	87.0	90.6				
Use of gas for cooking:						
Yes	2.4	1.7	1.4 (0.5–4.5)			
No	97.6	98.3				
Prematurity:						
Yes	6.0	5.0	1.6 (0.7–3.7)			
No	96.0	95.0				
Exclusive breastfeeding for at least 3 months:						
Yes	75.5	63.4	1.7 (1.2–2.6)		1.5 (1.0–2.3)	
No	24.5	36.6				
Education level of caretaker						
Primary	54.2	52.8	1.1 (0.8–1.5)			
Post–primary	45.8	47.2				

*COR Crude Odds ratio, CI Confidence Interval,

†AOR Adjusted Odds ratio.

## Discussion

In this paper we discuss findings of our study of factors associated with asthma and bronchiolitis among children less than five years attending Mulago National referral hospital. The results show that genetic factors, prematurity and socioeconomic status play a significant role in development and/or exacerbation of asthma in young children in Uganda. Secondly, there are similarities in factors associated with asthma and bronchiolitis, suggesting common etiological and risk factors, and this may influence the designing of preventive interventions for both diseases.

### Genetic factors

There was a significant association between a history of maternal asthma and asthma in the patient. This is in conformity with many previous studies that have demonstrated an association between maternal asthma and development of childhood asthma and further emphasizes the important role of genetic susceptibility in development of asthma [5,20]. The precise mechanism for this is not clearly understood, but several workers have postulated that multiple genes are involved in pathogenesis of asthma such as; production of allergen-specific Immunoglobulin E(IgE), expression of broncho hyper-responsiveness, generation of inflammatory mediators such as cytokines and chemokines, and modulation of Th2 response to antigens [25-27]. These genes differ between ethnic groups [27]. Studies to identify the genes that influence development of asthma in Uganda are recommended. Identification of asthma susceptibility genes may be vital in asthma prevention and effective therapy.

A positive history of allergies such as rhinitis and eczema in the patient, which signifies possible atopy, was significantly associated with asthma. In addition, three-quarters of the participants were from urban settings, a factor that has been linked to development of atopy [9]. A study by Addo-Yobo and colleagues that compared rural and urban school children with asthma showed a higher prevalence of atopy among the urban rich children [2]. These findings affirm the role of atopy in development of childhood asthma. In this study, atopy was elicited through taking the history and clinical examination, but no allergology tests (skin prick tests and measurement of allergen-specific IgE) were done. Hence, it is evident that there is need for more extensive research on atopy and asthma among under-fives in this environment.

### Environmental factors

Use of gas stoves for cooking was associated with asthma. However, there was no significant association between asthma and other factors related to air pollution such as use of charcoal or firewood as cooking fuel. This finding is different from what earlier studies had found: a significant relationship between asthma and air pollution [28]. These findings may be explained by the fact that in Kampala, cooking using gas is done in-doors whereas charcoal stoves and firewood are used outdoors. Sometimes stoves and firewood are used in a smaller separate house designated as a ‘kitchen’, where children under five years have limited or no access. It is possible that this lowers the risk of getting into contact with significant amounts of fumes and hence development of asthma in our setting. In Uganda, use of gas for cooking is usually limited to affluent families [24] and this might partly explain the small number of caretakers in this study who use gas for cooking. This may also be an indirect indicator of the link between high socioeconomic status and asthma, which has been described in Africa [2].

In the current study, a high level of education of the caretaker was associated with asthma. Level of education was used as a crude indicator of socioeconomic status. Researchers in developed countries have shown that asthma is associated with low socioeconomic status [29,30]. However, in low income countries, the prevalence of asthma is higher among the affluent [2,31,32]. This is thought to be due to adapting the Western life styles where children are exposed to allergens, infections, motor vehicle pollution and irritants, from early infancy [2].

There was no association between exposure to tobacco smoke and childhood asthma in this study population. Previous studies have documented an association between asthma and exposure to tobacco smoke, particularly maternal smoking [5]. It is not clear why our study did not find a similar association. It might be due to the fact that the participants with history of exposure to tobacco smoke were few making it difficult to detect any association.

The protective role of exclusive breastfeeding against asthma was not demonstrated in this study population. Although some studies have documented the importance of exclusive breastfeeding in protecting against development of asthma, evidence to the contrary exists [33]. Studies in the industrialized nations have shown that the role of breastfeeding in prevention of asthma mainly applies to children with genetic susceptibility to asthma such as those with asthmatic mothers, rather than the general population [11,12,34]. Studies specifically focusing on the role of exclusive breastfeeding in prevention of asthma in Africa are recommended, given its benefits as a child survival strategy especially in infection prevention and control, and nutrition, factors which are closely linked to development of asthma. This would provide more evidence on the benefits of exclusive breastfeeding in prevention of non-communicable diseases.

There was no association between childhood asthma and other factors like presence of pets and cockroaches in the households. Previous studies found an association between allergens like house-dust mite and animal dander and, development and exacerbation of asthma symptoms [35]. It is not clear why there was no association between allergens and asthma in this study. Further research on the role of allergens in asthma exacerbations is recommended. This would contribute towards designing asthma prevention strategies.

### Comparison of factors associated with asthma and bronchiolitis

The association between genetic factors such as a history of maternal asthma and allergy in the study participants, and asthma was significant. We observed a similar trend when we looked at factors associated with bronchiolitis, even though the association was marginally significant. Male gender was strongly associated with bronchiolitis. Similar findings were obtained in studies that looked at childhood asthma and genetic factors and, male gender [2,36]. These findings support the argument that, among young children, the distinction between asthma and bronchiolitis is not easily discernible [13]. The similarities regarding factors associated with asthma and bronchiolitis demonstrate the possibility of common aetiological and risk factors for both diseases [37,38].

There was no association between environmental factors such as exposure to tobacco smoke, prematurity and exclusive breastfeeding and bronchiolitis. Earlier studies indicated that the above factors play a significant role in development of bronchiolitis [15]. The reasons for the negative findings in this study are not clear. Further research to describe the relationship between bronchiolitis and asthma among young children in Uganda is recommended.

## Methodological considerations and limitations

### Study definitions

The study definitions were based on international guidelines and adapted to low-income settings and target age group. Other studies defined asthma based on previous history of cough and difficulty in breathing, and current/previous history of audible wheeze [39,40]. However, understanding of the concept of wheeze varies between caretakers and audible wheeze can only be recognized in only 30% of children with auscultatory wheeze [41,42]. Therefore, using a history of audible wheeze alone to define asthma might have excluded children in whom the audible wheeze was not recognized. The asthma case definition that was used in this study contributed to better identification of children with asthma, who would otherwise have been missed using a history of audible wheeze alone.

### Diagnostic approach

In this study, the diagnosis of asthma was based on stringent case definitions. The participants’ case reports were subjected to a detailed discussion by a panel of paediatricians with experience in respiratory conditions. The chest radiographs were interpreted by two independents radiologists. Therefore, we believe that this process increased the accuracy of the diagnoses hence providing more accurate estimates. However, the panel reviewed the case reports post hoc and did not have an opportunity to examine the patients. Therefore, the discussions on the clinical findings depended entirely on the record by the study doctor. Any errors she/he may have made during his/her observations could not be corrected, thus leading to some errors in diagnosis. Such errors were presumed to be minimal because the study doctors were trained and closely supervised by the principal investigator.

Respiratory infections can be caused by many viruses including Respiratory Syncytial Virus (RSV), Rhinoviruses, Adenoviruses and metapneumoviruses. In Africa, RSV is the most common cause of acute lower respiratory infections [43]. In this study, we analyzed the nasopharyngeal swab for RSV only, which contributes about one-third of respiratory viruses [43,44]. Hence, we might have missed out the children who had other viruses. The direct immunoflouroscence antibody (DFA) test, which has an estimated sensitivity of 83% and compares favourably with other techniques like Enzyme-linked Immunosorbent Assay (EIA), was used to identify RSV in the nasopharyngeal epithelium [45]. This approach may have identified most of the children with RSV infection.

Even though a history of maternal asthma was used in the case definition for asthma, it was used in combination with other criteria. We believe that the findings of an association between history of maternal asthma and childhood asthma were not substantially affected by the fact that it was part of the criterion for defining asthma.

We recruited only children with acute asthma symptoms. Hence, some of the factors that were found to be associated with asthma, such as use of gas for cooking, may have been triggers for the exacerbation rather that development of asthma. Finally, this was a tertiary hospital-based study which creates selection bias. We focused on children who had acute respiratory symptoms, leaving out those with chronic symptoms of asthma such as night coughs. These results can only be applied to similar health care settings.

## Conclusions

The study results show that atopy, prematurity and socioeconomic status, are important factors in development and/or exacerbation of asthma symptoms in young children. There is need for deeper exploration of the above factors in relation to childhood asthma to generate information that can be used in asthma prevention and control. Furthermore, the genetic susceptibility to bronchiolitis is similar to that of asthma. There is need for research to understand the link between bronchiolitis and asthma among young children in Africa.

## Competing interests

The authors declare that they have no competing interests.

## Authors’ contributions

RN, MSO and JKT participated in the conception and design of the study, with RN taking a lead role. RN participated in collection, analysis and interpretation of data. RN drafted the manuscript. GN, MSO and JKT reviewed the manuscript. All authors read and approved the final manuscript.

## Authors’ information

JKT is professor of Paediatrics and Child Health in the department of Paediatrics and Child Health, Makerere University College of Health Sciences, Kampala Uganda. GN is professor of Paediatrics and Child Health at Makerere College of Health Sciences Kampala Uganda.

MSO is a professor at the Research Unit for General Practice and Section of General Practice, Department of Public Health, University of Copenhagen, Denmark. RN is a PhD fellow and is the principal investigator in this study.

## Pre-publication history

The pre-publication history for this paper can be accessed here:

http://www.biomedcentral.com/1471-2431/13/141/prepub

## Supplementary Material

Additional file 1 Questionnaire used to elicit factors associated with asthma among children less than five years. Click here for file
