# Supplementary material for: Factors associated with asthma among under-fives in Mulago hospital, Kampala Uganda: a cross sectional study
Source: BMC Pediatr. 2013 Sep 11;13:141. doi: 10.1186/1471-2431-13-141 (PMC3848829; doi:10.1186/1471-2431-13-141)
Supplement: Additional file 1 — Questionnaire used to elicit factors associated with asthma among children less than five years. [file 1471-2431-13-141-S1.pdf]

1. QUESTIONNAIRE

Patient Identification

Date (dd/mm/yy)		
Study number		
Date of birth /age (***months***)		
Sex ***(Tick)***	Male	
	Female	
Address	District	
	Telephone contact	
Tick whichever is applicable	Urban	Rural

PART I:

**A**	**Your child's disease. How did it begin:**	Yes	No	Don't Know
1	For how long has your child been sick? **(days)**			
2	Did the disease start gradually over some days?			
3	Did the disease start with cold or nose symptoms?			
4a	Did the disease specifically start with much cough? If yes,			
b	i) Was the cough dry? **OR**			
	ii) Was the cough wet/productive?			
4c	Did the cough gradually worsen over some days?			
4d	Was the cough worse at night/early morning?			
5	Did your child have chest pain?			
6a	Did the disease specifically start with changes in breathing? If yes,			
6b	i) Difficulty in breathing?			
	ii) Fast/rapid breathing?			
	iii) Rattling/gurgling?			

	iv) Wheezing/whistling?			
	v) Shortness of breath?			
7	Did he/she have fever?			

B	**Your child's disease today:**	Yes	No	Don't Know
8	Does your child have;			
9a	Cough? If yes,			
9b	Is the cough dry **OR**			
9c	Is the cough wet/productive?			
10	Fast/rapid breathing?			
11	Difficulty in breathing?			
12	Wheezing/whistling?			
13	Rattling/gurgling?			
14	Fever?			

C	**In the last 3 months before coming to hospital, has your child:**	Yes	No	Don't Know
15	Had many colds?			
16a	Been coughing most of the time? If yes,			
16b	Coughed a lot in the night/early morning?			
16c	Woken up from sleep because of coughing?			
17	Had difficulty in breathing?			
18	Fast/rapid breathing?			
19	Wheezing/whistling?			
20	Rattling/gurgling?			
21	Shortness of breath			

22	Chest pain			
23a	Used any medicine because of cough? If yes,			
23b	Salbutamol			
23c	Steroids: Predinisolone, Dexamethasone, others (specify)			
23d	Cough syrups (specify)			
23	Antibiotics (specify)			

**D**	**Earlier problems (since birth):**	Yes	No	Don’t Know
	Has your child had recurrent episodes of:			
24a	Cough? If yes,			
24b	Is the cough mostly dry? **OR**			
24c	Is the cough wet/productive?			
24d	Does the cough usually occur in the night/early morning?			
24e	Does your child wake up because of cough?			
25a	Difficulty in breathing? If yes,			
25b	Did the difficulty in breathing usually occur in the night/early morning?			
25c	Did your child wake up because of difficulty in breathing?			
26a	Wheezing/whistling? If yes,			
26b	Did the wheezing usually occur in the night/early morning?			
26c	Did your child wake up because of wheezing?			
27	How old was your child when he/she started having cough problems?	Years/months		
28	Has your child been diagnosed with asthma before?			
29a	Has your child ever taken medicine for asthma or wheezing? If yes,			
29b	Salbutamol(oral, nebulised, inhaler)			
29c	Steroids(Predinisolone, Hydrocortisone, Dexamethasone, others-specify)			

PART II: FACTORS ASSOCIATED WITH ASTHMA

30a	Does your child have/ever had any allergies? If yes,	Yes	No	Don't Know
30b	i) Allergic rhinitis?			
	ii) Eczema?			
	iii) Allergic conjunctivitis?			
	iv) Others (specify)			
31	Are there any family members who have/have ever had asthma? If yes,			
31b	i) Mother			
	ii) Father			
	iii) Siblings			
	iv) Other (specify)			
32a	Are there any family members who have/have had allergies? If yes			
32b	i) Mother			
	ii) Father			
	iii) Siblings			
	iv) Other (specify)			

	**Birth history:**	Yes	No	Don't Know

33	Mode of delivery Spontaneous Vaginal Delivery			
	Caesarean section			
34	Maturity of baby			
	Pre-term			
	Term			
	Post-term			
35	Birth weight (kg)			
	i) ELBW (less than 1.5kg)			
	ii) LBW (1.5 – 2.5kg)			
	iii) Average (2.5 – 4.0kg)			
	iv) Big baby (more than 4 kg)			

	**Feeding:**	Yes	No	Don't Know
36a	Did your child ever breastfeed? If yes, ***(if No, go to 37)***			
36b	For how long?			
	i) Still breastfeeding			
	ii) < 3 months			
	iii) 3-6 months			
	iv) 6-12 months			
	v) > 12 months			
36c	For how long was your child exclusively breastfed?			
	i) Still exclusively breastfeeding			
	ii) < 3 months			
	iii) 3-6 months			

	**Environment:**	Yes	No	Don't

				Know
37a	How many people do live in your household?			
37b	Do you have a carpet in your house? If yes, what type?			
	i) Woolen			
	ii) Plastic			
	iii) Others (specify)			
37c	Do you have any rodents/pests in your house? If yes,			
	i) Cockroaches			
	ii) Mice			
	iii) Other (specify)			
37d	What do you use (fuel) for cooking?			
	i) Wood			
	ii) Charcoal			
	iii) Gas			
	iv) Electricity			
	v) Kerosene			

	**Exposure to tobacco smoke:**	Yes	No	Don't Know
38a	Is there any smoker at home? If yes, who?			
38b	i) Mother			
	ii) Father			
	iii) Other (specify)			
38c	How much does he/she smoke?			
	i) Light (less than 5 sticks per day)			
	ii) Moderate (5-20 sticks per day)			
	iii) Heavy (more than 20 sticks per day)			
38d	Does the child share a bedroom with the smoker?			

39	**Socioeconomic characteristics: Level of education of caretaker**	Yes	No	Don't Know
	i) No formal education			
	ii) Primary			
	iii) Secondary			
	iv) Tertiary			

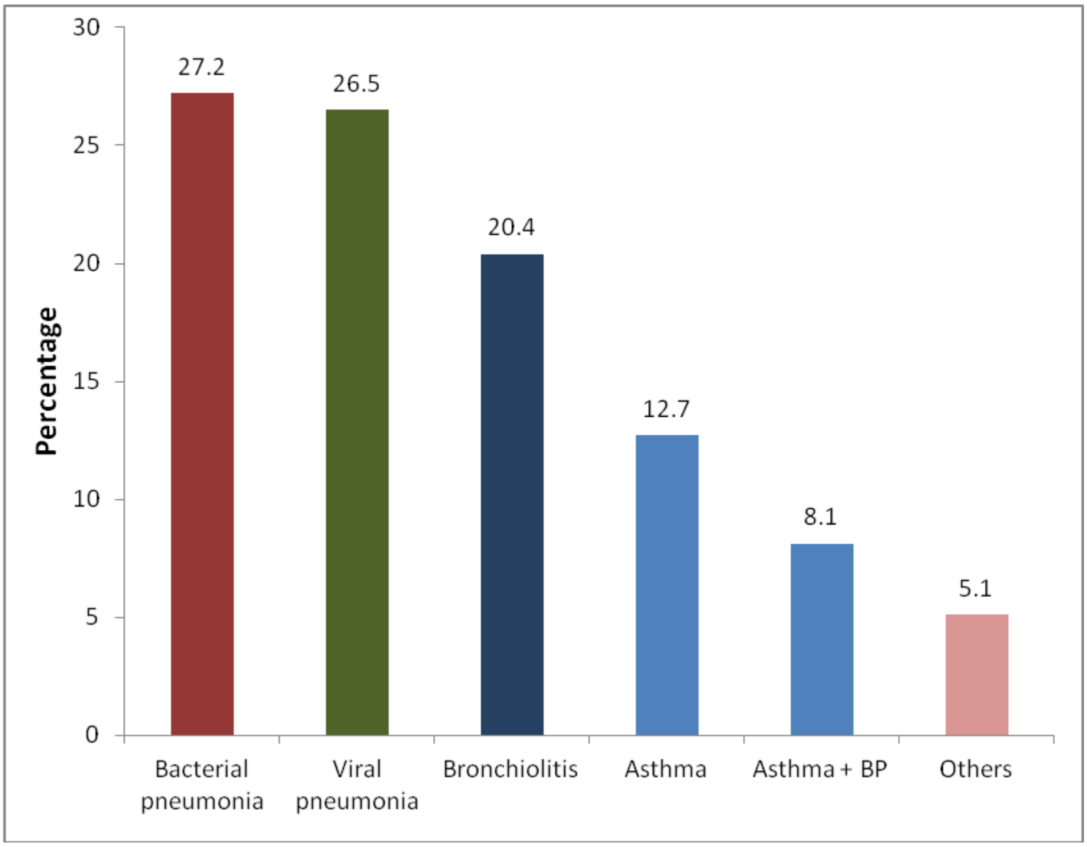

BP: bacterial pneumonia   Others included pulmonary tuberculosis and PCP.
